# Hematologic secondary malignancies among 102 Chinese patients with Waldenstrom’s macroglobulinemia: a single-center case experience and literature review

**DOI:** 10.3389/fonc.2023.1280033

**Published:** 2023-11-27

**Authors:** Lingling Wang, Conglin Xi, Yongfen Huang, Hao Xu, Yuqing Miao, Yuexin Cheng

**Affiliations:** ^1^Department of Hematology, The First People’s Hospital of Yancheng, The Yancheng Clinical College of Xuzhou Medical University, Yancheng, China; ^2^Department of Oncology, The Second People’s Hospital of Huai’an, The Affiliated Huaian Hospital of Xuzhou Medical University, Huaian, China

**Keywords:** Waldenstrom’s macroglobulinemia, secondary malignancies, B cell, incidence, prognosis, Chinese

## Abstract

Waldenstrom’s macroglobulinemia (WM) is a rare and indolent B-cell lymphoma. To investigate the type and survival of hematologic secondary malignancies (SMs) in Chinese patients with WM, we retrospectively reviewed the characteristics of 102 patients with WM from February 2002 to May 2023 in our center. Four men and two women were diagnosed with hematologic SMs. Of the six patients with hematologic SMs, one was diagnosed with acute myeloid leukemia (AML), one with multiple myeloma (MM), one with myelodysplastic syndrome (MDS), one with B-cell acute lymphoblastic leukemia (B-ALL), and two with diffuse large B-cell lymphoma (DLBCL). The median age was 65.5 years (56–74 years). The median interval time between diagnosis of WM and hematologic SMs was 39.5 months (10–117 months). Among those with WM with hematologic SMs, five died and one survived. Overall survival (OS) was just 33 months (12–119 months) on median. A total of 32 patients died and 64 survived in the group of WM without hematologic SMs, and the median OS was 82 months (3–250 months). This is the first study in the Chinese population on hematologic SMs in WM. The purpose of this study was to investigate the prognosis of hematologic SMs in WM in the Chinese population, as well as to compare the population’s characteristics to those of other centers. We investigated the underlying causes further and presented a research strategy for our forthcoming investigation. We intend to investigate risk factors for SMs as well as more accessible screening methods.

## Introduction

Waldenstrom’s macroglobulinemia (WM) is a rare and indolent lymphoma characterized by monoclonal IgM (1). MYD88L265P and CXCR4 mutations are common molecular alterations that can affect the efficacy and progression-free survival (PFS) of WM patients (2, 3). The indolent course of WM and the emergence of new drugs have led to prolonged survival in more patients with WM (4). Concomitantly, the phenomenon of secondary malignancies (SMs) is becoming more pronounced. Few studies have demonstrated the risk of hematologic SMs in WM, with the types being predominantly aggressive lymphomas and acute leukemia (5–8). The subjects of these studies were mostly European and American populations, the onset of disease was mainly from 1970 to 2010, and the treatment options were predominantly alkylating agents or nucleoside analogs. It remains controversial whether conventional and new drug-based combination therapy induces hematologic SMs in the WM population, and if there are differences in the prognosis of WM and the incidence of hematologic SMs between the two regimens.

Most importantly, there are no reports of hematologic SMs in WM in the Chinese population. Our study is the first retrospective study of hematologic SMs in WM in the Chinese population. The time frame of the study was from 2002 to 2023, and the treatment regimen was not only limited to alkylating agents and nucleoside analogs, with 55% of the patients receiving treatment based on rituximab, proteasome inhibitors, and BTK inhibitors (BTKi). Our study aimed to analyze the type and prognosis of hematologic SMs in WM in the Chinese population, as well as to compare the characteristics of the population with those of other centers. We further analyzed the potential pathogenesis and proposed the research plan for our subsequent research.

## Materials and methods

### Diagnostic criteria

All patients met the diagnostic criteria for WM (9): i) monoclonal IgM of any concentration; ii) small B lymphocytes, plasmacytoid lymphocytes, and plasma cells involving the bone marrow; iii) immunophenotypes indicated positive for CD19, CD20, sIgM, CD22, CD25, CD27, FMC7, CD38, and/or CD138 and negative for CD5, CD10, CD23, and CD103. However, 10% to 20% of patients may also express CD5, CD10, or CD23. Any other small B-cell lymphoid neoplasm that may also have plasmacytic differentiation is excluded. Hematologic SMs were confirmed by bone marrow aspiration and histopathology.

### Patients

A total of 102 patients diagnosed with WM from February 2002 to May 2023 were included in this study, excluding patients who were lost to follow-up. Based on the diagnostic criteria, they were divided into two groups: six patients with hematologic SMs and 96 patients without hematologic SMs. The diagnosis was confirmed by histopathology and cytopathology in all patients. This study was approved by the ethics committee of The First People’s Hospital of Yancheng, and all patients provided informed consent according to the Declaration of Helsinki.

### Data collection

Baseline clinical data from 102 cases, including gender, age, blood routine, biochemical routine, immunofixation electrophoresis, IgM level, therapy regimen, time to progression, and survival time, were collected from the hospital-based service and via telephone follow-up.

## Results

### Patients’ characteristics at diagnosis

A total of 102 patients with WM were included in this study. **Table 1** presents the baseline characteristics of the patients. There were 84 men and 18 women, with a male/female ratio of 4.67. The median age was 66 years (47–86 years). There were 45 patients aged ≤65 years, 38 patients aged 65–75 years, and 19 patients aged ≥75 years. Thirty-six patients presented with B symptoms. Sixty-one patients had revised International Prognostic Scoring System (r-IPSS) scores <3. Ninety-six patients had hemoglobin <115 g/L, and 24 patients had platelets <100 × 10^9^/L. Twelve patients had lactate dehydrogenase (LDH) >250 U/L. Forty-three patients had β2 microglobulin (β2-MG) >4 mg/L. Albumin was <35 g/L in 79 cases. Hepatomegaly was found in 16 cases, splenomegaly in 12 cases, and lymph node enlargement in 48 cases. A total of 80 patients underwent MYD88L265P testing, of whom 71 were positive and nine were negative. There were 54 cases with a previous history of cardiovascular and cerebrovascular diseases. By the end of the follow-up, there were 37 deaths and 65 survivors. The median overall survival (OS) was 72 months (3–250 months).

**Table 1:** Characteristics of 102 patients with WM at diagnosis.

Characteristics	Enrolled patient (n = 102)	WM with hematologic SMs (n = 6)	WM without hematologic SMs (n = 96)
Gender			
Male	84	4 (67%)	80 (83%)
Female	18	2 (33%)	16 (17%)
Age			
≤65 years	45	3 (50%)	42 (44%)
65–75 years	38	3 (50%)	35 (36%)
≥75 years	19	0	19 (20%)
With B symptoms	36	3 (50%)	33 (34%)
r-IPSS score			
0	5	1 (17%)	4 (4%)
1	23	2 (33%)	21 (22%)
2	33	0	33 (34%)
3	27	3 (50%)	24 (25%)
4	13	0	13 (14%)
5	1	0	1 (1%)
WBC ≤ 4×10^9^/L	34	3 (50%)	31 (32%)
Hb < 115 g/L	96	6 (100%)	90 (94%)
PLT < 110 × 10^9^/L	24	2 (33%)	22 (23%)
LDH > 250 U/L	12	0	12 (13%)
β2-MG < 4 mg/L	43	3 (50%)	40 (42%)
Albumin < 35 g/L	79	5 (83%)	74 (77%)
Hepatomegaly	16	1 (17%)	15 (16%)
Splenomegaly	12	0	12 (13%)
Lymphadenopathy	48	3 (50%)	45 (47%)
MYD88L265P			
Positive	72	6 (100%)	66 (69%)
Negative	9	0	9 (9%)
Not tested	21	0	21 (22%)
Previous history of cardiovascular and cerebrovascular diseases	54	1 (17%)	53 (55%)
Utilization of alkylating agents and nucleoside analogs			
Yes	46	5 (83%)	41 (43%)
No	56	1 (17%)	55 (57%)
Status			
Survival	65	1 (17%)	64 (67%)
Death	37	5 (83%)	32 (33%)
Median OS (months)	72 (3–250)	33 (12–119)	82 (3–250)

OS, overall survival; r-IPSS, revised International Prognostic Scoring System; WM, Waldenstrom’s macroglobulinemia; WBC, white blood cell; Hb, hemoglobin; PLT, platelet; LDH, lactate dehydrogenase; β2-MG, β2 microglobulin.

### Characteristics of six WM with hematologic SMs

Six patients had hematologic SMs: four men and two women. The median age was 65.5 years (56–74 years). Of the six patients with hematologic SMs, one was diagnosed with acute myeloid leukemia (AML), one with multiple myeloma (MM), one with myelodysplastic syndrome (MDS), one with B-cell acute lymphoblastic leukemia (B-ALL), and two with diffuse large B-cell lymphoma (DLBCL). The median interval time between diagnosis of WM and hematologic SMs was 39.5 months (10–117 months). The characteristics of the six patients with WM with hematologic SMs are listed in **Table 2**.

**Table 2:** Characteristics of six WM with hematologic SMs.

Case	Gender	Age (year)	Diagnosis of hematologic SMs	The interval time between WM and hematologic SMs (months)	Therapeutic regimen before hematologic SMs	Status (cause of death)	OS (months)
1^a^	Male	65	AML Karyotypic: 35~44, XY, del(5)(q31), del(7)(q22), dic(9;17)(q11;p11), add(11)(q25), add(12)(p11), add(13)(p11), del(16)(q22), -18, -20, -21, +1~2mar, inc[cp20]	10	COP, Fludarabine, RCP	Died (pulmonary infection)	12
2	Male	69	MM (IgM-Kapaa, DS: Phase III Group B, ISS: Phase III) Karyotypic: 46, XY [7] FISH: RB1(−), D13S319(−), 1q21(−), P53(+), IgH(+)	23	FC	Died (gastrointestinal bleeding, MODS)	27
3	Male	66	MDS (EB-2) r-IPSS: extremely high-risk group Karyotypic: 46, XY, der(18;21)(q10;q10), +21[19]/46, XY [1] NGS: DNMT3A, RUNX1	32	Fludarabine, BR	Died (infectious shock, pulmonary infection)	33
4	Female	74	B-ALL Karyotypic: 45, XX, add (4)(q31), -9 [15] NGS: KMT2D, MECOM	47	Bortezomib, BTKi	Died (pulmonary infection)	52
5	Male	56	DLBCL (non-GCB, Phase IV Group B)	60	CHOP	Died (chylothorax, respiratory failure)	61
6	Female	59	DLBCL (non-GCB, Phase III Group B) Bcl-2 and Myc double expression Karyotypic: 46, XY [20]	117	FC	Survived	119

AML, acute myeloid leukemia; BR, bendamustine and rituximab; B-ALL, B-cell acute lymphoblastic leukemia; CHOP, cyclophosphamide, doxorubicin, vincristine, and prednisone; COP, cyclophosphamide, vincristine, and prednisolone; DLBCL, diffuse large B-cell lymphoma; DS, Durie–Salmon stage; FC, fludarabine and cyclophosphamide; r-IPSS, revised International Prognostic Scoring System; MDS, myelodysplastic syndrome; MM, multiple myeloma; MODS, multiorgan dysfunction; NGS, next-generation sequencing; ISS, International Staging System; OS, overall survival; RCP, rituximab, cyclophosphamide, and prednisone; WM, Waldenstrom’s macroglobulinemia; SMs, secondary malignancies; BTKi, Bruton tyrosine kinase inhibitors; GCB, germinal center B cell; (-), negative; (+), positive.

a His brother was diagnosed with WM at the age of 70 years, with a history of dilated cardiomyopathy, and he died of heart failure in the 4th month.

### Clinical outcome of hematologic SMs in WM

In the group of WM with hematologic SMs, five patients died and one survived. The causes of death were mostly related to respiratory infections. The median OS was only 33 months (12–119 months). In the group of WM without hematologic SMs, 32 patients died and 64 survived; the median OS was 82 months (3–250 months).

Case 1 was a 65-year-old man who received multiple lines of therapy with vincristine, cyclophosphamide, and rituximab. The patient developed AML 10 months later, and no abnormalities in the fusion gene or mutation were detected. However, the chromosome showed 35~44, XY, del(5)(q31), del(7)(q22), dic(9;17)(q11;p11), add(11)(q25), add(12)(p11), add(13)(p11), del(16)(q22), -18, -20, -21, +1~2mar, inc[cp20]. The patient’s disease progressed rapidly, and he died of a pulmonary infection 2 months later. The overall survival was only 12 months. It is noteworthy that the patient’s brother was also diagnosed with WM at the age of 70 years. Because of a previous history of dilated cardiomyopathy, he died of heart failure 4 months later. Case 2 was a 69-year-old man with secondary MM at the 23rd month who was treated with the fludarabine and cyclophosphamide (FC) regimen only. Fluorescence *in situ* hybridization (FISH) detected TP53 mutation and IgH rearrangement. It has been demonstrated that the TP53 mutation is related to a lower survival rate in MM patients (10–12). In the 27th month, this patient died due to gastrointestinal bleeding and multiorgan dysfunction (MODS). The hematologic SMs in most WM were aggressive lymphoma and AML, with the third case developing high-risk MDS at the 32nd month. The chromosome was 46, XY, der(18;21)(q10;q10), +21 [19]/46, XY [1], with DNMT3A and RUNX1 mutations detected by next-generation sequencing (NGS). Pre-exposed drugs were fludarabine, bendamustine, and rituximab. The patient was treated with a course of venetoclax and azacitidine and achieved complete remission (CR) of the bone marrow and chromosomes, but no hematologic remission was obtained. The persistent hypoproliferative state of the bone marrow made it difficult to control the pulmonary infection, and he eventually died of infectious shock. The patient survived only 1 month after diagnosis of secondary MDS. Case 4 was diagnosed with WM at the age of 74 years, and because of poor physical status and advanced age, she was treated with low-dose bortezomib and later maintained with zanubrutinib. During follow-up, CT suggested enlarged retroperitoneal lymph nodes, and pathology tests were suggestive of B-ALL. The chromosome indicated 45, XX, add (4)(q31), -9 [15], and NGS suggested KMT2D and MECOM mutations. The patient received one cycle of VP (vindesine + prednisone) in combination with zanubrutinib and achieved partial remission (PR). However, due to poor physical status and recurrent lung infections, she eventually died from the infection. Cases 5 and 6, both secondary to DLBCL (non-germinal center B-cell-like (non-GCB)), were treated with alkylating agents or nucleotide analogs. MYD88L265P was positive in both cases. Unfortunately, we did not investigate the clonal evolution of the WM transition to DLBCL in any further detail. Emerging technologies should be able to solve this problem, such as single-cell RNA-seq. Case 5, a 56-year-old man, progressed to DLBCL 5 years after he was diagnosed with WM. He died 1 month after the diagnosis of DLBCL due to a combination of chylothorax and respiratory failure. Case 6 was a woman diagnosed with DLBCL (double expression of Bcl-2 and Myc) at the 117th month, with no adverse mutations found during NGS testing, and was alive by the endpoint of observation.

## Discussion

Several studies demonstrated an increasing risk of hematologic SMs in lymphoproliferative disorder (LPD) (13–16). However, there are very few studies on hematologic SMs in WM. Since WM is an indolent lymphoma, it requires quite a long follow-up period. To the best of our knowledge, this is the first study to date on hematologic SMs in WM in the Chinese population. We included 102 consecutive patients in our center who all had indications for treatment, with six (5.88%) cases diagnosed with hematologic SMs. Previous studies are controversial as to whether the choice of treatment regimen has an impact on survival status and the risk of hematologic SMs. Compared with those in published studies, 55% of the patients in our center received new drug-based treatment regimens, which is the most important difference from the reported studies.

The common denominator of these five deaths was rapid disease progression and very short survival. The causes of death were mostly related to infections and complications. Among the cases included in the study, 54 (53%) patients had a previous history of chronic cardiovascular and cerebrovascular diseases such as hypertension, coronary heart disease, type 2 diabetes, and cerebral infarction. After receiving chemotherapy and immune-targeted therapy, these patients often have difficulty controlling various types of infections or complications, which is a big challenge when managing elderly patients with WM.

We summarize and compare published studies of hematologic SMs in WM (**Table 3**). There are currently four large-sample studies published in 2011, 2012, and 2015. These studies were predominantly conducted in European and American populations, with a time frame from 1973 to 2011. The types of hematologic SMs were predominantly DLBCL and AML. The median time from diagnosis of WM to hematologic SMs was 50 months.

**Table 3:** Comparison of different studies on WM with hematologic SMs.

Study	Time of publication	Period	Country	Total number of cases	Type of hematologic SMs (number)	The median interval time between diagnosis of WM and hematologic SMs (months)	Risk factors	Reference
Steven P. Treon et al.	2011	1999–2010	Boston	20/924 (2.1%)	Myeloid leukemia (4) B-cell malignancies (13) T-cell malignancies (2) Other (1)	NA	Familial predisposition	5
R. P. Ojha et al.	2012	1973–2008	SEER-9	45/1,618 (2.7%)	AML (6) NHL (30) Myeloma (9)	NA	NA	6
Varettoni et al.	2011	1980–2009	Pavia, Milan, and Italy	10/230 (4.3%)	DLBCL (6) AML (3) CML (1)	49 (24–145)	Previously treated patients	7
Jorge J. Castillo et al.	2015	1992–2011	SEER-13	166/4,676 (3.5%)	Lymphomas (120) Aggressive NHL (39) Indolent NHL (47) Other (34) Myeloma (31) Acute leukemia (15)	51 (19–96)	Age, female, prolongation of the disease	8

SEER, Surveillance Epidemiology and End Results program; NA, not applicable; WM, Waldenstrom’s macroglobulinemia; SMs, secondary malignancies; AML, acute myeloid leukemia; NHL, non-Hodgkin’s lymphoma; DLBCL, diffuse large B-cell lymphoma; CML, chronic myelogenous leukemia.

Jorge J. Castillo et al. (8) used the SEER-13 database to analyze the characteristics of 174 hematologic SMs among 4,676 cases of WM, concluding that the incidence of SMs was independent of age, sex, and year of diagnosis but related to disease transformation, therapeutic regimen, and immune dysregulation. The cumulative incidence of hematologic SMs in WM at 5 and 10 years was 2.3% (95% CI, 1.9%–2.8%) and 4.2% (95% CI, 3.5%–4.9%), respectively. For the first time, the risk of hematologic SMs was compared between WM and the general population. Patients with WM had a fourfold higher risk of hematologic SMs. Compared to the general population, the risk of secondary DLBCL was 4.33 (95% CI, 2.94–6.15), and for AML, it was 3.21 (95% CI, 1.79–5.39). As for the causes of hematologic SMs, the study believed that the occurrence of AML was associated with the therapeutic regimen, while aggressive lymphomas were related to Richter transformation. As for indolent lymphoma and myeloma, the cause is unknown. Although there was no statistical difference in the occurrence of SMs by sex, a significantly higher risk of secondary lymphoma and myeloma was observed in women than in men. This study suggested that aging with WM rather than aging may be a driving factor in the induction of SMs in WM. With prolonged exposure to various therapeutic agents, there is an increased risk of receiving antigenic stimulation and immune dysregulation. Previous studies by Jorge J. Castillo et al. (17) found that first-degree relatives of patients with WM had a 20% risk of being diagnosed with WM and other hematologic malignancies, which suggested that genetically inherited susceptibility may be a factor of hematologic SMs in WM. However, the study also had some limitations, most notably that it did not include an analysis of the treatment regimen’s effect on survival.

In a retrospective study of 230 WM, M. Varettoni, et al. (7) found that 10 (4%) had hematologic SMs, of whom six were DLBCL, three were MDS/AML, and one was chronic myelogenous leukemia (CML). The median time of WM to hematologic SMs was 49 (24–145) months. The cumulative risk of hematologic SMs at 10 and 15 years was 6% and 8%, respectively. Although the risk of hematologic SMs was not statistically different in treatment and exposure to alkylating agents and nucleoside analogs, the study still found that five of the six patients with secondary DLBCL had been exposed to alkylating agents, and all three patients with secondary MDS/AML had been exposed to nucleoside analogs. The risk of hematologic SMs in treated WM patients was four times higher than that in untreated patients. As the study was conducted in patients with WM diagnosed between 1980 and 2009, the majority of therapeutic agents were alkylating agents, and the proportion of newer drug-based regimens, such as rituximab, proteasome inhibitors, and BTKi, was very low. Due to the short follow-up, it remains to be verified whether the treatment regimen has an impact on the development of hematologic SMs in a larger sample and a longer follow-up. In 2012, R. P. Ojha (6) commented on Varettoni’s study as small in sample size, so they retrospectively analyzed 1,618 WM patients registered in the SEER database from 1973 to 2008. A total of 45 patients had hematologic SMs, of whom 30 were non-Hodgkin’s lymphoma (NHL), nine were myeloma, and six were AML.

A study published in 2011 by Steven P. Treon et al. (5) retrospectively analyzed 924 patients with WM, focusing on whether familial predisposition had an adverse effect on SMs. There were 20 (2.16%) cases with hematologic SMs in WM. Among them, 92.4% of the events occurred in patients with a family history of WM.

The published studies were dominated by European and American populations before 2011 and did not include the effect of treatment regimens on prognosis and the risk of developing SMs. However, our study was conducted in the Chinese population in the time frame of 2003 to 2023, and the treatment regimen changed from traditional chemotherapy to regimens dominated by new agents such as rituximab, bortezomib, and BTKi. Our earlier work found that WM is characterized by familial co-morbidity, especially in first-degree relatives (18). We followed up on these patients and found that the proportion of hematologic SMs was not high, but the survival of this subgroup of patients was significantly lower than that of sporadic WM.

However, there are some limitations to our study. First, owing to the small sample size, it was not possible to perform a subgroup analysis of patients with hematologic SMs, especially the baseline data such as age and gender. It is challenging to depict the cumulative incidence of SMs in this cohort due to the statistically significant distinction in case numbers between these two groups in our center. Furthermore, as there are currently only six cases of WM with hematologic SMs in our center, it is not possible to compare the spectrum of this study with patients from Western countries. This problem will be solved in our next study. We plan to expand the sample size by including primary and recurrent WM cases from all the major hematology centers in Jiangsu Province, China. We will also set the time frame as the last 20 years to analyze whether patients in different years affect the incidence of hematologic SMs. Based on our previous work, we found that the proportion of Chinese patients with familial WM was high, and this subgroup will be followed up and compared with the other centers to verify whether there are differences in survival and risk of hematologic SMs in different races. Second, it is difficult to achieve deep remission with traditional chemotherapy regimens, while newer drugs such as BTKi show a higher degree of remission. However, long-term maintenance therapy is required, and there is no indication of discontinuation. Fifty-five percent of the patients in this study received new drug-based combination therapy, and the current follow-up is still short. The off-target effect and drug resistance of BTKi still have not been adequately resolved, and whether the long-term use of new drugs increases the risk of hematologic SMs remains to be answered by longer follow-up. Furthermore, 81 (79%) patients in this study were tested only for the MYD88 mutation. Whole exome sequencing (WES) was performed in only nine patients, two of whom were with hematologic SMs. We expect the next phase of the study to perform WES in more cases to explore whether there are potential driver genes that can predict the occurrence of SMs. In addition, due to the high cost of WES, which greatly increases the financial burden of patients, we are also exploring whether we can monitor the tumor load by detecting measurable residual disease (MRD) in patients with WM and analyze if there is an association between MRD and the incidence of SMs.

Overall, this is the first study to date on hematologic SMs in WM in the Chinese population. Our work found that the median survival time of WM with hematologic SMs (33 months) was significantly lower than that of general WM (82 months). The next study is aimed at exploring the predictive factors associated with the incidence of SMs as well as more accessible screening methods.

## Data availability statement

The original contributions presented in the study are included in the article/supplementary material. Further inquiries can be directed to the corresponding authors.

## Ethics statement

The studies involving humans were approved by The First People’s Hospital of Yancheng. The studies were conducted in accordance with the local legislation and institutional requirements. The participants provided their written informed consent to participate in this study. Written informed consent was obtained from the individual(s) for the publication of any potentially identifiable images or data included in this article.

## Author contributions

LW: Conceptualization, Data curation, Formal analysis, Investigation, Methodology, Project administration, Resources, Software, Supervision, Validation, Visualization, Writing – original draft, Writing – review & editing. CX: Conceptualization, Data curation, Formal analysis, Investigation, Methodology, Project administration, Resources, Software, Supervision, Validation, Visualization, Writing – original draft, Writing – review & editing. YH: Formal analysis, Funding acquisition, Methodology, Project administration, Resources, Supervision, Validation, Visualization, Writing – review & editing. HX: Conceptualization, Formal analysis, Funding acquisition, Investigation, Methodology, Project administration, Resources, Supervision, Validation, Visualization, Writing – review & editing. YM: Conceptualization, Data curation, Formal analysis, Funding acquisition, Investigation, Methodology, Project administration, Resources, Software, Supervision, Validation, Visualization, Writing – original draft, Writing – review & editing. YC: Conceptualization, Data curation, Formal analysis, Funding acquisition, Investigation, Methodology, Project administration, Resources, Software, Supervision, Validation, Visualization, Writing – original draft, Writing – review & editing.
